# Longitudinal variation in muscle strength and mobility in patients in an intensive care unit: a retrospective cohort study

**DOI:** 10.62675/2965-2774.20260229

**Published:** 2026-01-28

**Authors:** Liana Accioly Melo Habib, Larissa Laranjeira Pinheiro dos Santos, Isabel Lisboa Santiago Nascimento, Thaysa Vitorio de Lima, Yone Kauane da Silva Lima, Manuella Franco Cerqueira da Silva, Dimitri Gusmao-Flores, Bruno Prata Martinez

**Affiliations:** 1 Universidade Federal da Bahia Hospital Universitário Professor Edgard Santos Salvador BA Brazil Hospital Universitário Professor Edgard Santos, Universidade Federal da Bahia - Salvador (BA), Brazil.; 2 Universidade Federal da Bahia Department of Physiotherapy Salvador BA Brazil Department of Physiotherapy, Universidade Federal da Bahia - Salvador (BA), Brazil.; 3 Universidade Federal Bahia Salvador BA Brazil Postgraduate Program in Medicine and Health, Universidade Federal Bahia - Salvador (BA), Brazil.

**Keywords:** Intensive care units, Mobility limitation, Muscle strength, Muscle weakness

## Abstract

**Objective:**

To longitudinally evaluate muscle strength and mobility in an intensive care unit and identify factors associated with muscle weakness at intensive care unit discharge.

**Methods:**

A retrospective cohort study was conducted with patients who had their muscle strength measured at some point during their intensive care unit stay. Muscle strength was assessed using the Medical Research Council score, and measurements were taken at two points: as soon as medically possible (first assessment) and discharge. Mobility was assessed using the Functional Status Score for the intensive care unit scale, which includes bed transfer and locomotion activities. These activities were evaluated at three points: previous status, as soon as medically possible (first assessment), and discharge.

**Results:**

The change in muscle strength in the sample of 1,310 patients between the assessment at discharge [56 (48 - 60)] and the first assessment [54 (48 - 60)] was significant (p value < 0.001). When comparing mobility levels, a significant difference (p < 0.001) was observed between the time prior to hospitalization [35 (34 - 35)], the first assessment [28 (20 - 33)], and discharge [29 (21 - 35)]. Factors associated with muscle weakness were length of stay in the intensive care unit [OR 1.16 (1.06 - 1.28); p = 0.002]; use of sedation [OR 3.8 (1.27 - 11.16); p = 0.016] and muscle strength score at the first assessment [OR 0.84 (0.79 - 0.90); p = 0.001].

**Conclusion:**

Muscle strength and mobility increased from the first assessment to discharge. Prospective studies are needed to explore the trends observed in this study.

## INTRODUCTION

The development of intensive care unit-acquired weakness (ICU-AW) is one of the main complications of critical illness, with a prevalence of 45% as described in a recent systematic review.^(1)^ This weakness can persist between 6 months and 2 years after discharge from the ICU, with 29% of patients not recovering within 5 years.^(2)^ It predisposes patients to complications in the short term, such as increased time on mechanical ventilation, length of stay in the ICU/hospital, ICU/hospital mortality, and hospital costs. In the long term, it includes increased post-ICU mortality, reduced functionality, and a greater likelihood of long-term care in rehabilitation centers.^(2)^

Regarding functional status, there is also an association between low functionality and increased post-discharge mortality. Furthermore, patients whose functional status improved before discharge had a reduced odds ratio for mortality after discharge.^(3)^ Due to its relevance, the assessment of functional skills pre-ICU, upon admission, and at discharge from the ICU has been recommended as a strategy to identify and manage deficiencies to optimize rehabilitation.^(4)^

Knowing the importance of these variables as measures of functionality and their relationship with adverse outcomes, as well as the possibility of carrying out preventive and rehabilitation interventions by the multidisciplinary team, it is essential to understand the changes in muscle strength and mobility during ICU stay. Furthermore, there is a lack of published data that evaluates muscle strength and mobility longitudinally in the ICU. Therefore, the objective of the present study was to to longitudinally evaluate muscle strength and mobility in an ICU and identify factors associated with muscle weakness at ICU discharge.

## METHODS

### Study design, setting, and participants

This is an observational retrospective cohort study, with data obtained from electronic medical records from the multidisciplinary team at the ICUs of the *Hospital Universitário Professor Edgard Santos* (HUPES), which comprises two adult units, each with ten beds. Intensive care unit 1 has a general clinical patient population, while ICU 2 specializes in cardiovascular and neurological patients. The research was approved by the HUPES Research Ethics Committee (CEP/HUPES) and followed the Strengthening the Reporting of Observational studies in Epidemiology (STROBE) recommendations.^(5)^ The study included all patients admitted to both adult ICUs at HUPES from April 2019 to May 2022 who had their muscle strength measured at some point during their ICU stay. Patients who died while in the ICU and with an ICU stay ≤ 2 days were excluded.

### Variables

The variables studied were age, sex, length of stay in the ICU, clinical or surgical profile, use of non-invasive ventilation (NIV), orotracheal intubation, duration of mechanical ventilation (MV), outcomes related to weaning (accidental extubation, extubation failure, percentage of tracheostomy), previous mobility, time to first sedestration, time to first orthostasis, time to first ambulation, use of vasoactive drugs, sedoanalgesia, neuromuscular blocker, dialysis, and Acute Physiology and Chronic Health Disease Classification System (APACHE II). Data on muscle strength at the first assessment (as soon as medically possible) and at discharge from the ICU were analyzed, as well as the respective percentages of muscle weakness at both times (first assessment and discharge from the ICU). Data on mobility prior to hospitalization, at the first assessment, and at discharge from the ICU were also analyzed.

### Data sources/measurement

Data collection was performed by the physical therapy team of the aforementioned units, with muscle strength being assessed on two occasions (first assessment and discharge) and mobility on three occasions (previous state, first assessment, and discharge). The criteria for the first assessment of muscular strength were the patient's adequate level of cooperation and clinical and cardiovascular stability (respiratory rate < 35 breaths per minute; systolic blood pressure between 90 and 180mmHg; mean arterial pressure between 60 and 110mmHg; heart rate between 40 and 130 beats per minute; peripheral oxygen saturation > 90%; no reports of respiratory discomfort and pain; in addition to the absence of other contraindications for performing exercises with the upper and lower limbs). To assess manual muscle strength, the Medical Research Council (MRC) score was used, which measures the bilateral strength of 12 muscle groups of the upper limbs (shoulder abductors, elbow flexors, and wrist extensors) and lower limbs (hip flexors, knee extensors, and ankle dorsiflexors), bilaterally. Each muscle group is scored on a zero to five point scale, with a total score of zero to 60; a value lower than 48 indicates muscle weakness.^(6)^ Mobility was assessed using the Brazilian version of the Functional Status Scale in the ICU (FSS-ICU), which assesses the movements of rolling over in bed, transferring from a supine to a sitting position, transferring from a sitting to a standing position, sitting at bedside, and walking, with a total score that ranges from zero to 35.^(7)^ The value considered as reduced mobility was an FSS < 30.

In these ICUs, the physical therapy team works full-time every day (24 hours/day) and receives in-service training to apply the MRC and FSS instruments to monitor patients’ muscular strength and mobility performance. They provided a progressive mobilization plan based on the patients’ clinical and functional condition, as discussed during a multidisciplinary visit, focusing on exercises, transfer training, verticalization, and ambulation. In these units, patients also undergo weaning protocols with daily assessment of sedoanalgesia and spontaneous breathing test according to criteria.

### Statistical analysis

The numerical variables were described as means and standard deviations when normally distributed, medians and interquartile ranges when abnormally distributed, and as percentages for categorical variables. To compare muscle strength values between discharge and the first assessment, the non-parametric Wilcoxon paired test was performed. To compare mobility at the three measurement points (previous, first assessment, and discharge), a nonparametric repeated-measures test was used. A backward stepwise logistic regression was also performed to evaluate factors associated with muscle weakness and mobility reduced at the time of ICU discharge, for variables that had a p value < 0.1, through the inclusion of continuous (age, ICU length of stay, MV time, APACHE II, body mass index, score and time for 1 MRC assessment) and categorical (clinical profile, neuromuscular blocker, NIV, sedation, vasoactive drugs, dialysis) variables. The p value considered statistically significant was < 0.05. The data were analyzed using the JAMOVI program, version 2.5.2, which is open-access software.

## RESULTS

**Figure 1:**
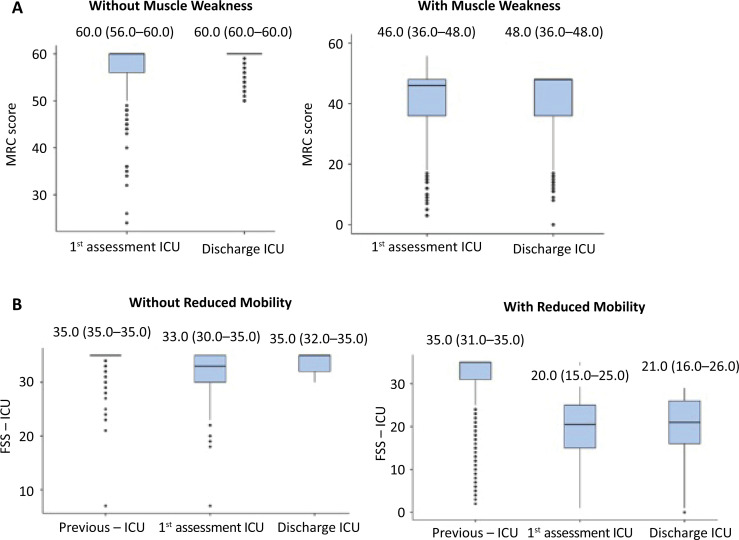
Description of variation in muscle strength; (A) one assessment and discharge from the intensive care unit, with and without muscle weakness at intensive care unit discharge, and mobility; (B) previous state, one assessment and discharge from the intensive care unit, with and without reduced mobility at intensive care unit discharge, during intensive care unit hospitalization.

The final sample consisted of 1,310 patients, whose muscle strength and mobility were measured at the first assessment and at discharge. Tables 1 and 2 present the general characterization of the studied sample, and flowchart (Figure 1S - Supplementary Material) describes patients evaluated in the different moments, as well as the percentage of deaths in the units, which was 9.1%. The frequency of muscle weakness was 45.6% (590) at the first assessment and 42.5% (550) at discharge. Regarding mobility, at the time of the previous status assessment, 12.4% (162) had reduced mobility (FSS < 30); at the first assessment, this reduction was 58.5% (766); and at discharge, it was 50.1% (656).

**Table 1:** Descriptive data of the sample of patients included in the study, stratified into those with and without muscle weakness at discharge from the intensive carer unit (n = 1,295)

		n = 1,295	Muscle weakness (n = 550)	No muscle weakness (n = 745)	p value
Age (years)		58.0 (44.0 - 68.0)	60.0 (48.0 - 69.0)	56.0 (43.0 - 67.0)	< 0 .001
Surgical profile		560 (43.2)	207 (37.6)	353 (47.4)	< 0.001
Reason for ICU admission					
	Cardiovascular	413 (31.9)	147 (26.7)	266 (35.7)	< 0.001
	Neurological	214 (16.5)	94 (17.4)	120 (16.1)	0.087
	Gastrohepatic	90 (6.9)	44 (8.0)	46 (6.2)	0.916
	Oncohematological	64 (4.9)	31 (5.6)	33 (4.4)	0.901
ICU length of stay (days)		5.0 (2.0 - 8.0)	7.0 (4.0 - 11.0)	4.0 (3.0 - 6.0)	< 0.001
BMI (kg/m^2^)		24.1 (20.8 - 27.9)	23.5 (19.8 - 27.3)	24.8 (21.6 - 28.5)	< 0.001
APACHE II		14.0 (10.0 - 19.0)	16.0 (12.0 - 21.0)	13.0 (10.0 - 17.0)	< 0.001
Women		676 (52.2)	325 (59.1)	351 (47.1)	0.336
Orotracheal intubation (yes)		283 (21.8)	182 (33.1)	101 (13.6)	< 0.001
MV time (days)		1.2 (0.3 - 3.1)	1.8 (0.6 - 4.6)	0.4 (0.2 - 1.4)	< 0.001
Extubation time (days)		1.1 (0.3 - 3.1)	1.7 (0.6 - 4.0)	0.4 (0.2 - 1.3)	< 0.001
Extubation failure		19 (6.7)	17 (3.0)	2 (0.2)	0.016
Accidental extubation		9 (3.2)	8 (1.4)	1 (0.1)	0.118
Tracheostomy		39 (13.8)	28 (5.2)	11 (1.4)	0.214
NIV (yes)		69 (5.3)	41 (7.4)	28 (3.7)	0.003
NIV failure (yes)		14 (20.2)	12 (2.1)	2 (0.2)	0.025
Sedation (yes)		287 (22.2)	187 (34.0)	100 (13.)	<0.001
Neuromuscular blocker (yes)		8 (0.6)	6 (1.1)	2 (0.2)	0.062
Vasoactive drugs (yes)		567 (43.8)	275 (50.0)	292 (39.1)	< 0.001
Dialysis (yes)		56 (4.3)	23 (4.1)	33 (4.4)	0.006
MRC score - first assessment		54.0 (48.0 - 60.0)	46.0 (36.0 - 48.0)	60 (56.0 - 60.0)	< 0.001
LOS during first MRC assessment		1.0 (0.0 - 1.8)	1.0 (0.0 - 2.0)	0.0 (0.0 - 1.0)	< 0.001
MRC score - discharge		56.0 (48.0 - 60.0)	48.0 (36.0 - 48.0)	60.0 (60.0 - 60.0)	< 0.001
LOS during discharge MRC assessment		4.0 (3.0-7.0)	5.0 (3.0 - 8.0)	4.0 (3.0 - 5.0)	< 0.001
FSS score - previous		35.0 (34 - 35.0)	35.0 (30.0 - 35.0)	35.0 (35.0 - 35.0)	< 0.001
FSS score - first assessment		28.0 (20.0 - 33.0)	21.0 (15.0 - 27.0)	31.0 (27.0 - 35.0)	< 0.001
FSS score - discharge		29.0 (21.0 - 35.0)	21.0 (15.0 - 28.0)	33.0 (29.0 - 35.0)	< 0.001
Sedestration (yes)		1,294 (99.9)	549 (99.8)	745 (100.0)	0.244
Ambulation (yes)		875 (67.6)	257 (46.7)	618 (82.9)	< 0.001
Time - first sitting (days)		2.0 (1.0 - 3.0)	2.0 (1.0 - 4.0)	1.0 (1.0 - 2.0)	< 0.001
Time - first orthostasis (days)		2.0 (1.0 - 4.0)	3.0 (2.0 - 6.0)	2.0 (1.0 - 4.0)	< 0.001
Time - first ambulation (days)		3.0 (1.0 - 4.0)	3.0 (2.0 - 6.0)	2.0 (1.0 - 4.0)	< 0.001

ICU - intensive care unit; BMI - body mass index; APACHE - Acute Physiology and Chronic Health Evaluation; MV - mechanical ventilation; NIV - non-invasive ventilation; MRC - Medical Research Council; LOS - length of stay; FSS - Functional Status Score. Values are reported as median (interquartile range 25 - 75%) and as numbers (percentages).

**Table 2:** Descriptive data of the sample of patients included in the study, stratified into those with and without reduced mobility at discharge from the intensive care unit (n =1,310)

		n = 1,310	Reduced mobility (n = 656)	No reduced mobility(n = 654)	p value
Age		58.0 (44.0 - 68.0)	61.0 (48.0 - 70.0)	56.0 (42.0 - 65.0)	< 0.001
Surgical profile		564 (43.0)	262 (39.9)	302 (46.2)	0.023
Reason for ICU admission					
	Cardiovascular	415 (31.7)	43 (6.5)	372 (56.9)	0.014
	Neurological	218 (16.6)	22 (3.3)	196 (30.0)	0.379
	Gastrohepatic	91 (6.9)	12 (1.8)	79 (12.1)	0.530
	Oncohematological	67 (5.1)	8 (1.2)	59 (9.0)	0.807
ICU length of stay (days)		3.0 (1.0 - 5.0)	6.0 (4.0 - 10.3)	4.0 (3.0 - 6.0)	< 0.001
BMI		24.1 (20.8 - 27.9)	23.6 (20.3 - 27.5)	24.7 (21.4 - 28.1)	< 0.001
APACHE II		14.0 (10.0 - 19.0)	15.0 (11.0 - 20.0)	13.0 (10.0 - 18.0)	0.009
Women		686 (52.3)	367 (55.9)	319 (48.8)	0.073
Orotracheal intubation (yes)		286 (21.8)	193 (29.4)	93 (14.2)	0.009
MV time (days)		1.2 (0.3 - 3.6)	1.8 (0.6 - 4.7)	0.4 (0.2 - 1.4)	0.020
Extubation time (days)		1.1 (0.3 - 3.1)	1.7 (0.5 - 3.9)	0.4 (0.2 - 1.3)	0.017
Extubation failure		19 (6.2)	16 (2.4)	3 (0.4)	0.045
Accidental extubation		9 (2.7)	5 (0.7)	4 (0.6)	0.609
Tracheostomy		40 (13.9)	28 (4.2)	12 (1.8)	0.525
NIV (yes)		70 (5.2)	45 (6.8)	25 (3.8)	0.015
NIV Failure (yes)		14 (17.5)	12 (1.8)	2 (0.3)	0.061
Sedation (yes)		289 (22.0)	200 (30.5)	89 (13.6)	< 0.001
Neuromuscular blocker (yes)		8 (0.6)	7 (1.0)	1 (0.1)	0.034
Vasoactive drugs (yes)		575 (43.8)	337 (51.3)	238 (36.4)	< 0.001
Dialysis (yes)		56 (4.2)	37 (5.6)	19 (2.9)	0.015
MRC score - first assessment		54.0 (48.0 - 60.0)	48.0 (36.0 - 58.0)	60 (48.0 - 60.0)	< 0.001
LOS during first MRC assessment		1.0 (0.0 - 1.8)	1.0 (0.0 - 2.0)	0.0 (0.0 - 1.0)	0.024
MRC score - Discharge		56.0 (48.0 - 60.0)	48.0 (39.0 - 54.0)	60.0 (60.0 - 60.0)	< 0.001
LOS during discharge MRC assessment		4.0 (3.0 - 7.0)	5.0 (3.0 - 8.0)	4.0 (3.0 - 5.0)	0.161
FSS score - previous		35.0 (34.0 - 35.0)	35.0 (30.0 - 35.0)	35.0 (35.0 - 35.0)	< 0.001
FSS score - first assessment		28.0 (20.0 - 33.0)	20.0 (15.0 - 25.0)	33.0 (30.0 - 35.0)	< 0.001
FSS Score - discharge		29.0 (21.0 - 35.0)	21.0 (15.0 - 26.0)	35.0 (32.0 - 35.0)	< 0.001
Sedestration (yes)		1309 (99.9)	655 (99.8)	654 (100.0)	0.318
Ambulation (yes)		881 (67.2)	232 (35.4)	649 (99.2)	< 0.001
Time – first sitting (days)		2.0 (1.0 - 3.0)	2.0 (1.0 - 4.0)	1.0 (1.0 - 2.0)	0.006
Time - first orthostasis (days)		2.0 (1.0 - 4.0)	3.0 (1.0 - 6.0)	2.0 (1.0 - 3.0)	0.136
Time - first ambulation (days)		3.0 (1.0 - 4.0)	3.0 (2.0 - 6.0)	2.0 (1.0 - 4.0)	0.077

ICU - intensive care unit; BMI - body mass index; APACHE - Acute Physiology and Chronic Health Evaluation; MV - mechanical ventilation; NIV - non-invasive ventilation; MRC - Medical Research Council; LOS - length of stay; FSS - Functional Status Score. Values are reported as median (interquartile range 25-75%) and as numbers (percentages).

The difference in muscle strength between the assessment at discharge [56 (48 - 60)] and the first assessment [54 (48 - 60)] was significant (p < 0.001). When comparing mobility levels, a p-value of < 0.001 was also observed between the time prior to hospitalization [35 (34 - 35)], the first assessment [28 (20 - 33)], and discharge [29 (21 - 35)]. The analysis of these variations in muscular strength and mobility, by covariate, is described in table 2 and figure 1.

MRC - Medical Research Council; FSS - Functional Status Score; ICU - intensive care unit.

In the analysis of factors associated with muscle weakness upon discharge from the ICU, the variables that were associated were length of stay in the ICU [OR 1.16 (1.06 - 1.28); p = 0.002], use of sedation [OR 3.8 (1.27 - 11.16); p = 0.016] and muscle strength score at the first assessment [OR 0.84 (0.79 - 0.90); p = 0.001] (Table 3). For reduced mobility, the associated factors were age [OR 1.03 (1.01 - 1.06); p = 0.019]; length of stay in the ICU [OR 1.13 (1.03 - 1.23); p = 0.007] and muscle strength score at the first assessment [OR 0.91 (0.86 - 0.95); p = 0.001] (Table 4). The collinearity analyses of the variables included in the logistic regression are described in tables 1S and 2S (Supplementary Material).

**Table 3:** Logistic regression analysis of factors associated with muscle weakness at intensive carer unit discharge (n = 1,295)

Variable	OR (95%CI)	p value
Age	0.99 (0.97 - 1.03)	0.708
ICU length of stay (days)	1.16 (1.06 - 1.27)	0.002
Clinical profile	0.84 (0.30 - 2.32)	0.739
MV time (days)	0.90 (0.74 - 1.08)	0.269
Sedation (yes)	3.87 (1.31 - 11.42)	0.014
Vasoactive drugs (yes)	0.40 (0.12 - 1.30)	0.136
Dialysis(yes)	0.66 (0.10 - 4.15)	0.656
APACHE II	1.05 (0.98 - 1.12)	0.163
MRC score first assessment (each MRC score point)	0.84 (0.79 - 0.90)	0.001
Time for 1 MRC assessment (days)	0.92 (0.78 - 1.08)	0.287
BMI	0.97 (0.89 - 1.05)	0.497
NIV	0.43 (0.07 - 2.51)	0.352
Neuromuscular blocker	0.31 (0.01 - 7.27)	0.470

ICU - intensive care unit; MV - mechanical ventilation; APACHE - Acute Physiology and Chronic Health Evaluation; MRC - Medical Research Council; BMI - body mass index; NIV - non-invasive ventilation.

**Table 4:** Logistic regression analysis of factors associated with mobility reduction at intensive carer unit discharge (n = 1,310)

Variable	OR (95%CI)	p-value
Age (years)	1.03 (1.01 - 1.06)	0.019
ICU length of stay (days)	1.13 (1.03 - 1.23)	0.007
Clinical profile	1.09 (0.42 - 2.83)	0.845
MV time (days)	1.03 (0.82 - 1.28)	0.830
Sedation(yes)	1.24 (0.46 - 3.13)	0.661
Vasoactive drugs (yes)	2.27 (0.83 - 6.19)	0.110
Dialysis(yes)	0.41 (0.08 - 2.14)	0.293
APACHE II	1.03 (0.97 - 1.09)	0.381
MRC score first assessment (each MRC score point)	0.91 (0.86 - 0.95)	0.001
Time for 1 MRC assessment (days)	0.88 (0.77 - 1.01)	0.073
BMI	0.99 (0.91 - 1.06)	0.712
NIV	0.63 (0.12 - 3.08)	0.566
Neuromuscular blocker	1.54 (0.09 - 26.02)	0.765

ICU - intensive care unit; MV - mechanical ventilation; APACHE - Acute Physiology and Chronic Health Evaluation; MRC - Medical Research Council; BMI - body mass index; NIV - non-invasive ventilation.

## DISCUSSION

In this study, significant differences in muscle strength were observed between the first assessment and discharge, as well as variation in mobility between the moments before the first assessment and discharge in the general sample. Factors associated with muscle weakness included length of stay in the ICU and sedation use, with muscle strength at the first assessment associated with protection. For reduced mobility upon discharge from the ICU, the factors were ICU length of stay and age, with muscle strength at the first assessment also associated with protection.

To our knowledge, this is the first study with a sample of more than 1,310 patients that evaluated the change in muscle strength and mobility throughout their ICU stay. The data identified an increase in muscle strength and mobility between the first assessment and discharge from the ICU. These changes in strength and mobility are indicators of team results and are monitored monthly. This monitoring is crucial due to the association of higher scores at discharge with better outcomes after hospital discharge.^(3,8)^

The percentage of patients with muscle weakness found at the two moments evaluated (first assessment and discharge) was equivalent the data described in the scientific literature,^(9–29)^ which can be primarily explained by the profile of patients included in the study, the average length of hospital stay of 5 days, the reduced time to first uprighting compared to previous studies,^(30,31)^ and the assessment instrument used to diagnose weakness. Regarding the instrument used, the literature reported a value of 43% (95%CI 31 - 55%) for muscle weakness in studies that used only the MRC for the diagnosis of weakness,^(1)^ which is equivalent to the value found in the present study. A recent observational cohort study, whose objective was to develop and validate an ICU-AW prediction model, used the MRC to assess muscle strength at bedside in 400 patients and reported an incidence of ICU-AW of 14.39% in the model group and 17.5% in the validation group, values similar to those found in this research.^(32)^ One aspect to be considered in our study population that may justify the difference in the percentage of weakness found was the fact that it included patients with a hospital stay of three days or more.

In relation to the MRC, despite being considered valid and viable for use in any ICU, as it does not require equipment and presents good intra- and inter-examiner reliability when used by trained therapists,^(6,14)^ it has the limitation of not necessarily being able to be used in the moment of admission to the ICU, as many patients were not able to respond to the commands necessary for measurement. Therefore, the prevalence of ICU-AW in this study may be underestimated. One way to improve precision in future studies may be to use non-volitional muscle-strength assessment methods, which can identify weakness earlier. However, these methods are more expensive, require specialized staff, and have their parameters still under validation.

Regarding mobility, this was also assessed in the first assessment, similar to muscular strength. An evaluation of prior mobility was collected through self-report from the patient and/or family members, which helps understand a possible decline between the moment prior to hospitalization, the first assessment, and discharge. The data identified a reduction in mobility between the previous moment and the first assessment, with a slight recovery to the ICU, but without reaching the state prior to admission, as shown in table 2. These data are similar to a recent prospective multicenter study conducted on patients diagnosed with COVID-19, which reported that 33.2% recovered functional independence. This study did not evaluate strength longitudinally, but identified that recovery of physical function at hospital discharge was associated with muscle strength at ICU discharge and length of stay in the ICU.^(31)^

When analyzing factors related to muscle weakness at discharge from the ICU, an association was observed with ICU length of stay [1.16 (1.06 - 1.27)] and sedation use, consistent with data from previous studies. This association can be justified by the longer exposure time to variables such as immobility, systemic inflammation, hyperglycemia, and MV.^(4,8–12,18–20,23,24,26,28,32)^ As a protective factor against muscle weakness, the variable was the muscle strength score at the first assessment. This protection must be analyzed with caution, since it is a retrospective study, and the muscle strength values between the two moments showed a small reduction.

The data were collected in two ICUs, one with a more clinical profile (non-surgical patients) and the other with a more surgical profile, with a predominance of patients with cardiovascular and neurological problems. These ICUs admit patients through the public health network across all regions of the state of Bahia, thereby strengthening external validity for other ICUs in Brazil.

### Study limitations

The study has limitations due to its retrospective nature, including the lack of data on the occurrence of *delirium*, hyperglycemia, parenteral nutrition, sepsis, and the amount of mobilization performed during hospitalization, all of which are related to muscle weakness. Another important limitation is that the study was conducted at a single center, limiting its generalizability. It is important to mention that all patients received daily mobilization according to the unit's routine, as well as being subjected to a daily sedation withdrawal protocol. Another limitation of the study was the measurement of muscular strength using the MRC scale, which may introduce some divergence, especially at levels four and five, as it is a categorical variable and may introduce measurement bias. However, this bias may have been minimized through training conducted with the team of professionals, who apply this instrument daily in the unit.

## CONCLUSION

Muscle strength and mobility increase from the first assessment to discharge. Factors associated with muscle weakness at discharge from the intensive care unit included length of stay and sedation use, with the muscle strength score from the first assessment showing a protective association. Prospective studies are needed to explore the trends observed in this study.

## Data Availability

The contents underlying the research text are included in the manuscript.

## References

[1] Fazzini B, Märkl T, Costas C, Blobner M, Schaller SJ, Prowle J (2023). The rate and assessment of muscle wasting during critical illness: a systematic review and meta-analysis. Crit Care.

[2] Vanhorebeek I, Latronico N, Van den Berghe G (2020). ICU-acquired weakness. Intensive Care Med.

[3] Rydingsward JE, Horkan CM, Mogensen KM, Quraishi SA, Amrein K, Christopher KB (2016). Functional status in ICU survivors and out of hospital outcomes: a cohort study. Crit Care Med.

[4] Mikkelsen ME, Still M, Anderson BJ, Bienvenu OJ, Brodsky MB, Brummel N (2020). Society of Critical Care Medicine's International Consensus Conference on Prediction and Identification of Long-Term Impairments After Critical Illness. Crit Care Med.

[5] von Elm E, Altman DG, Egger M, Pocock SJ, Gøtzsche PC, Vandenbroucke JP, STROBE Initiative (2007). Strengthening the Reporting of Observational Studies in Epidemiology (STROBE) statement: guidelines for reporting observational studies. BMJ.

[6] Hermans G, Clerckx B, Vanhullebusch T, Segers J, Vanpee G, Robbeets C (2012). Interobserver agreement of Medical Research Council sum-score and handgrip strength in the intensive care unit. Muscle Nerve.

[7] Silva VZ, Araújo JA, Cipriano G, Pinedo M, Needham DM, Zanni JM (2017). Brazilian version of the Functional Status Score for the ICU: translation and cross-cultural adaptation. Rev Bras Ter Intensiva.

[8] Dinglas VD, Aronson Friedman L, Colantuoni E, Mendez-Tellez PA, Shanholtz CB, Ciesla ND (2017). Muscle weakness and 5-year survival in acute respiratory distress syndrome survivors. Crit Care Med.

[9] de Letter MA, Schmitz PI, Visser LH, Verheul FA, Schellens RL, Op de Coul DA (2001). Risk factors for the development of polyneuropathy and myopathy in critically ill patients. Crit Care Med.

[10] Parry SM, El-Ansary D, Cartwright MS, Sarwal A, Berney S, Koopman R (2015). Ultrasonography in the intensive care setting can be used to detect changes in the quality and quantity of muscle and is related to muscle strength and function. J Crit Care.

[11] Van Aerde N, Meersseman P, Debaveye Y, Wilmer A, Gunst J, Casaer MP (2020). Five-year impact of ICU-acquired neuromuscular complications: a prospective, observational study. Intensive Care Med.

[12] Diaz Ballve LP, Dargains N, Urrutia Inchaustegui JG, Bratos A, Milagros Percaz M, Bueno Ardariz C (2017). Weakness acquired in the intensive care unit. Incidence, risk factors and their association with inspiratory weakness. Observational cohort study. Rev Bras Ter Intensiva.

[13] Nguyen The L, Nguyen Huu C (2015). Critical illness polyneuropathy and myopathy in a rural area in Vietnam. J Neurol Sci.

[14] Hough CL, Lieu BK, Caldwell ES (2011). Manual muscle strength testing of critically ill patients: feasibility and interobserver agreement. Crit Care.

[15] Brunello AG, Haenggi M, Wigger O, Porta F, Takala J, Jakob SM (2010). Usefulness of a clinical diagnosis of ICU-acquired paresis to predict outcome in patients with SIRS and acute respiratory failure. Intensive Care Med.

[16] Weber-Carstens S, Koch S, Spuler S, Spies CD, Bubser F, Wernecke KD (2009). Nonexcitable muscle membrane predicts intensive care unit-acquired paresis in mechanically ventilated, sedated patients. Crit Care Med.

[17] Ahlbeck K, Fredriksson K, Rooyackers O, Mäbäck G, Remahl S, Ansved T (2009). Signs of critical illness polyneuropathy and myopathy can be seen early in the ICU course. Acta Anaesthesiol Scand.

[18] Sharshar T, Bastuji-Garin S, Stevens RD, Durand MC, Malissin I, Rodriguez P (2009). Groupe de Réflexion et d'Etude des Neuromyopathies En Réanimation. Presence and severity of intensive care unit-acquired paresis at time of awakening are associated with increased intensive care unit and hospital mortality. Crit Care Med.

[19] Nanas S, Kritikos K, Angelopoulos E, Siafaka A, Tsikriki S, Poriazi M (2008). Predisposing factors for critical illness polyneuromyopathy in a multidisciplinary intensive care unit. Acta Neurol Scand.

[20] Ali NA, O'Brien JM, Hoffmann SP, Phillips G, Garland A, Finley JC (2008). Midwest Critical Care Consortium. Acquired weakness, handgrip strength, and mortality in critically ill patients. Am J Respir Crit Care Med.

[21] Latronico N, Bertolini G, Guarneri B, Botteri M, Peli E, Andreoletti S (2007). Simplified electrophysiological evaluation of peripheral nerves in critically ill patients: the Italian multi-centre CRIMYNE study. Crit Care.

[22] Amaya-Villar R, Garnacho-Montero J, García-Garmendía JL, Madrazo-Osuna J, Garnacho-Montero MC, Luque R (2005). Steroid-induced myopathy in patients intubated due to exacerbation of chronic obstructive pulmonary disease. Intensive Care Med.

[23] Bednarík J, Vondracek P, Dusek L, Moravcova E, Cundrle I (2005). Risk factors for critical illness polyneuromyopathy. J Neurol.

[24] Garnacho-Montero J, Amaya-Villar R, García-Garmendía JL, Madrazo-Osuna J, Ortiz-Leyba C (2005). Effect of critical illness polyneuropathy on the withdrawal from mechanical ventilation and the length of stay in septic patients. Crit Care Med.

[25] Bercker S, Weber-Carstens S, Deja M, Grimm C, Wolf S, Behse F (2005). Critical illness polyneuropathy and myopathy in patients with acute respiratory distress syndrome. Crit Care Med.

[26] De Jonghe B, Sharshar T, Lefaucheur JP, Authier FJ, Durand-Zaleski I, Boussarsar M (2002). Groupe de Réflexion et d'Etude des Neuromyopathies en Réanimation. Paresis acquired in the intensive care unit: a prospective multicenter study. JAMA.

[27] Druschky A, Herkert M, Radespiel-Tröger M, Druschky K, Hund E, Becker CM (2001). Critical illness polyneuropathy: clinical findings and cell culture assay of neurotoxicity assessed by a prospective study. Intensive Care Med.

[28] Garnacho-Montero J, Madrazo-Osuna J, García-Garmendia JL, Ortiz-Leyba C, Jiménez-Jiménez FJ, Barrero-Almodóvar A (2001). Critical illness polyneuropathy: risk factors and clinical consequences. A cohort study in septic patients. Intensive Care Med.

[29] Tepper M, Rakic S, Haas JA, Woittiez AJ (2000). Incidence and onset of critical illness polyneuropathy in patients with septic shock. Neth J Med.

[30] Morris PE, Goad A, Thompson C, Taylor K, Harry B, Passmore L (2008). Early intensive care unit mobility therapy in the treatment of acute respiratory failure. Crit Care Med.

[31] Stripari Schujmann D, Claudia Lunardi A, Neri Peso C, Pompeu JE, Annoni R, Miura MC (2022). Functional Recovery Groups in Critically Ill COVID-19 Patients and Their Associated Factors: From ICU to Hospital Discharge. Crit Care Med.

[32] Yang Z, Wang X, Chang G, Cao Q, Wang F, Peng Z (2023). Development and validation of an intensive care unit acquired weakness prediction model: a cohort study. Front Med (Lausanne).

