# Supplementary material for: Longitudinal variation in muscle strength and mobility in patients in an intensive care unit: a retrospective cohort study
Source: Crit Care Sci. 2026 Jan 28;38:e20260229. doi: 10.62675/2965-2774.20260229 (PMC12977221; doi:10.62675/2965-2774.20260229)
Supplement: Supplementary Material [file 2965-2774-ccsci-38-e20260229-suppl1.pdf]

Critical Care Science
https://doi.org/10.62675/2965-2774.20260229

SUPPLEMENTARY MATERIAL

Longitudinal variation in muscle strength and mobility in patients in an intensive care unit: a retrospective cohort study

Liana Accioly Melo Habib[1], Larissa Laranjeira Pinheiro dos Santos[2], Isabel Lisboa Santiago Nascimento[2], Thaysa Vitorio de Lima[2], Yone Kauane da Silva Lima[2], Manuella Franco Cerqueira da Silva[2], Dimitri Gusmao-Flores[3], Bruno Prata Martinez[3]

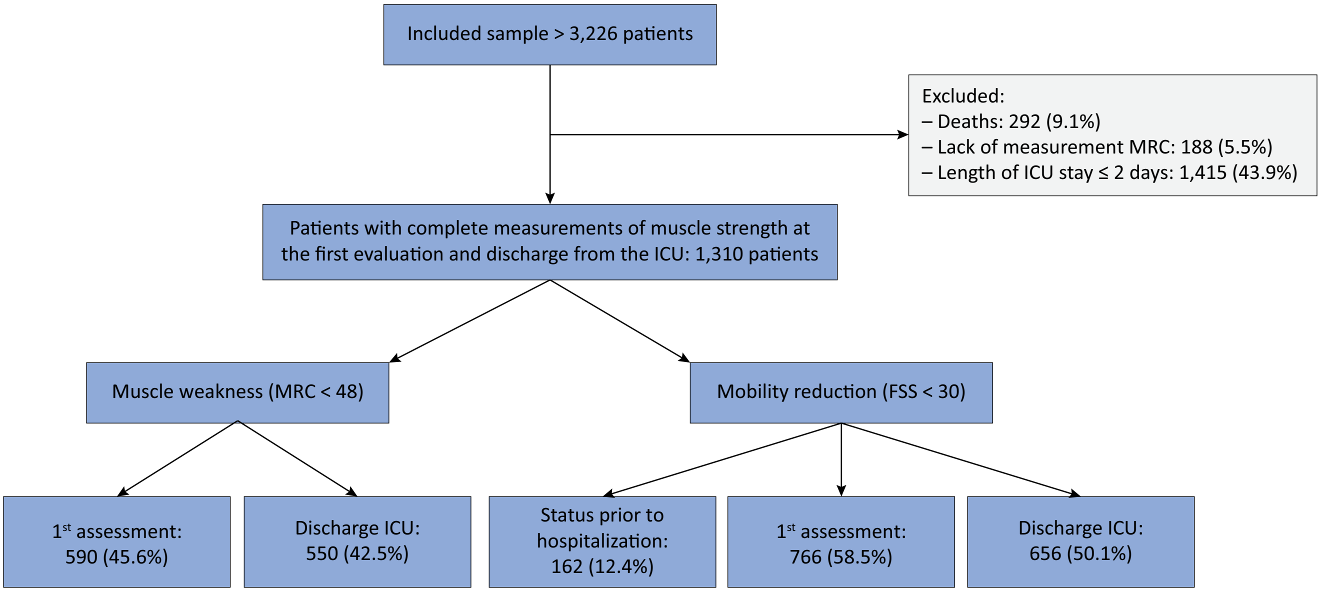

MRC - Medical Research Council; ICU - intensive care unit.

Figure 1S - Patient flow chart.

This is an open access article under the CC BY license (https://creativecommons.org/licenses/by/4.0/).

Table 1S - Collinearity statistics - factors associated with muscle weakness upon intensive care unit discharge

	VIF	Tolerance
Age	1.26	0.795
ICU length of stay	2.47	0.405
APACHE II	1.19	0.843
Clinical profile	1.34	0.744
Vasoactive drugs (yes)	1.13	0.882
Sedation (yes)	1.20	0.833
Dialysis (yes)	1.19	0.840
MRC score first assessment	1.42	0.704
Neuromuscular blocker	1.17	0.857
NIV	1.45	0.689
MV time (days)	2.23	0.448
BMI	1.13	0.882
Time for 1 MRC assessment (days)	1.43	0.699

VIF - variance inflation factor; ICU - intensive care unit; APACHE - Acute Physiology and Chronic Health Evaluation; MRC - Medical Research Council; NIV - non-invasive ventilation; MV - mechanical ventilation; BMI - body mass index.

Table 2S - Collinearity statistics - factors associated with reduced mobility upon intensive care unit discharge

	VIF	Tolerance
Age	1.21	0.827
ICU length of stay	1.90	0.525
MV time (days)	2.09	0.478
MRC score first assessment	1.33	0.754
BMI	1.11	0.897
APACHE II	1.24	0.809
Neuromuscular blocker	1.25	0.800
Clinical profile	1.36	0.736
NIV	1.31	0.764
Vasoactive drugs (yes)	1.09	0.917
Dialysis (yes)	1.26	0.791
Sedation (yes)	1.16	0.864
Time for 1 MRC assessment (days)	1.80	0.557

VIF - variance inflation factor; ICU - intensive care unit; MV - mechanical ventilation; MRC - Medical Research Council; BMI - body mass index; APACHE - Acute Physiology and Chronic Health Evaluation; NIV - non-invasive ventilation;
